# EPAS 1, congenital heart disease, and high altitude: disclosures by genetics, bioinformatics, and experimental embryology

**DOI:** 10.1042/BSR20182197

**Published:** 2019-05-10

**Authors:** Consolato Sergi

**Affiliations:** 1 National ‘111’ Center for Cellular Regulation and Molecular Pharmaceutics, Key Laboratory of Fermentation Engineering (Ministry of Education), Hubei University of Technology, Wuhan 430068, P.R. China; 2 Department of Orthopedics, Tianyou Hospital, Wuhan University of Science and Technology, Wuhan, Hubei, P.R. China; 3 Department of Laboratory Medicine and Pathology, University of Alberta, Edmonton, AB, Canada; 4 Stollery Children’s Hospital, University Alberta Hospital, Edmonton, AB, Canada

**Keywords:** congenital, EPAS1, heart

## Abstract

The high-altitude environment is a challenge for human settlement. Low oxygen concentrations, extreme cold, and a harsh arid climate are doubtlessly challenges for the colonization of the Tibetan plateau. I am delighted to comment on the article of Pan et al. (2018) on mutations in endothelial PAS domain-containing protein 1 (EPAS1) in congenital heart disease in Tibetans. In humans, the *EPAS1* gene is responsible for coding EPAS1 protein, an alias of which is HIF2α, an acronym for hypoxia-inducible factor 2 alpha. EPAS1 is a type of hypoxia-inducible factors, which are collected as a group of transcription factors involved in body response to oxygen level. *EPAS1* gene is active under hypoxic conditions and plays an essential role in the development of the heart and in the management of the catecholamine balance, mutations of which have been identified in neuroendocrine tumors. In this article, Pan et al. investigated Tibetan patients with and without non-syndromic congenital heart disease. They identified two novel *EPAS1* gene mutations, of which N203H mutation significantly affected the transcription activity of the vascular endothelial growth factor (VEGF) promoter, particularly in situations of hypoxia. VEGF is a downstream target of HIF-2 (other than HIF-1), and the expression levels of either HIF-1α or HIF-2α correlate positively to VEGF expression. Pan et al.’s data may be of incitement to further evaluate protein–protein interaction and using experimental animal models. Moreover, it may also be a stimulus for setting up genetic epidemiologic studies for other populations living at high altitudes.

Congenital heart disease (CHD) affects millions of individuals worldwide, including over one million children in the United States with about one-fourth of children born with CHD requiring intensive surgical intervention within the first year of life. Despite improved cardiac surgical procedures and rates of survival into adulthood, incidences that surpass 90% of children remain at risk for neurological injury and neurobehavioral challenges that pose a threat to the quality of life across the lifespan [1]. The high-altitude environment is a challenge for human settlement. Low oxygen concentrations, extreme cold, and a harsh arid climate are doubtlessly daily challenges for the colonization of the Tibetan plateau [2]. Hypoxia is a central key of several widespread human diseases, such as ischemic heart disease, pulmonary arterial hypertension (PAH), and stroke. CHD is an ongoing topic for populations living in high altitudes. The incidence of CHD in newborns at high altitude is about 20 times higher than neonates born at a low height, comprising left to right shunt defects and rarely complex CHD. Infants, aged 12–18 months, living in high altitude have an incidence of CHD about 10 times higher than infants living at low altitude and most importantly, about 8% of these patients develop PAH or death [3]. The Tibetan autonomous prefecture of Yushu (average elevation over 4000 m) and the independent Mongolian county of Henan (average height over 3600 m) in Huangnan show the highest prevalence of CHD [4]. Chun et al. investigated 84302 students from Nagqu, Tibet and found a prevalence rate of CHD of 0.5%, i.e., 1 case every 200 [5]. The most common defects were patent ductus arteriosus (PDA; about 2/3), atrial septal defects (ASDs; about 1/5), and ventricular septal defects (VSD; about 1/10) with the prevalence of CHD in girls being higher than in boys. The condition of high altitudes may also have some effect on two routine cardiac surgery operations, including Fontan and bidirectional Glenn’s anastomosis, although a sum of factors should probably be taken into account (e.g., age) [6–9]. The Fontan procedure comprises any surgical operation that results in the blood flow of systemic venous blood to the lungs without passing through a ventricle, while the bidirectional Glenn is a common surgical fashion of the second stage of the total cavopulmonary connection (TCPC) surgery where the end of the superior vena cava (SVC) is connected to the side of the pulmonary artery (PA).

In humans, the Cbp/p300-interacting transactivator 2 is a protein that is encoded by the *CITED2* gene located on chromosome 6. CITED2 gene is a cardiac transcription factor that plays a crucial role in the development of the embryonic cardiovascular system. Knock-out experiments of CITED2 in mice may result in several cardiac defects. In a study involving 187 unrelated Tibetans with CHD, Liu et al. found a novel mutation of CITED2 that enhanced the expression of vascular endothelial growth factor (VEGF) under the role of co-receptor hypoxia-inducible factor 1 alpha (HIF-1α) [10]. In this journal, in a recent issue, Pan et al. studied the endothelial PAS domain-containing gene 1 (*EPAS1*) in CHD in Tibetans [11]. A group of 286 Tibetan patients with non-syndromic CHD and 250 separate Tibetan healthy controls were engaged from Qinghai, China using Sanger DNA sequencing and confirming the novelty of identified variants by the examination of 1000G and ExAC databases of the human genome. Moreover, Pan et al. investigated the effect of *EPAS1* mutations on the transcription of its target gene, VEGF, by dual-luciferase reporter assay. The authors of the Qinghai High Altitude Medical Research Institute together with colleagues from the Center for Genetics, National Research Institute for Family Planning, and Graduate School of Peking Union Medical College, Beijing, China identified two novel *EPAS1* gene mutations (N203H and G724W) in two patients. Although the G724W is silent, the N203H mutation significantly affects the transcription activity of the VEGF promoter, specifically in the setting of hypoxia. There is an enhanced protein–protein interaction between EPAS1 and the proteins arising from endoglin 1 (*EGLN1*) or Von-Hippel–Lindau (*VHL*) genes. The endoglin gene (*EGLN1*), often known as *PHD2*, encodes an enzyme called prolyl hydroxylase domain 2 (PHD2). The *VHL* gene encodes a protein (pVHL) that functions as part of a complex called the VCB–CuL2 complex, which is formed by pVHL and the gene products of *elongin C, elongin B, Cul-2*, and *Rbx1*, which functions as a ubiquitin-protein ligase [12]. The alpha-subunits of the HIFs have been identified as targets for the VCB–CuL2 ubiquitin ligase. The authors suggest that *EPAS1* gene mutations may play an etiologic role in the development of Tibetan non-syndromic CHD.

However, genetic defects should not be considered as merely deterministic factors for a pathological condition [13,14]. The twin-reversed arterial perfusion (TRAP) sequence, or acardia, is the most severe lethal condition in monozygotic twinning. It is part of the twin–twin transfusion syndrome (TTTS), a subtype of monochorionic twin pregnancy, showing an extremely high pre- and perinatal morbidity and mortality [15,16]. In TTTS there is a net transfusion of whole blood from the umbilical artery of the donor twin to the umbilical vein of the recipient twin in a villous zone of overlapping perfusion. The transfusion of blood from the donor to the recipient via placental arterio-arterial anastomoses in monochorionic gestations is the supposed mechanism, resulting in the formation of a TRAP sequence [17]. TRAP sequence is mainly linked to hypoxia [18]. The lack of oxygen during early embryogenesis can induce severe disruptions of head–brain and heart formation. An oxygen deficiency due to TRAP may be responsible not only for the encephaloclastic (destructive) changes but also for the developmental arrest of the brain in the receiving twin. Developmental abnormalities may result either from genetic events (e.g., point mutations, aneuploidy) or from exogenous factors disrupting the healthy development of the embryo [19]. About exogenous factors, abnormalities are primarily dependent on the time of interference of the teratogenic injuries (‘noxa’) with typical development, which means that their effects are highly phase-specific [20–24]. Hypoxia has been demonstrated to be a very useful teratogen, causing disruption, particularly of neurulation, if it interferes with early stages of embryonic development [21,23–26]. Experimental studies performed in amphibian and chick embryos showed that hypobaric-mediated hypoxia determines disruptions of the head, brain, and heart predominantly. The most severe brain and head changes resulted if the hypoxia was induced at the beginning of gastrulation, i.e., before the onset of neurulation, when oxygen consumption is known to be exceptionally high [25]. In these experiments, the underlying developmental mechanisms responsible for the malformations occurring with hypoxia were multiple including (1) disturbance of the migration of blastema (altered ‘topogenesis’ of the German Embryological School according to Lehmann [27,28] and ‘integrated cell and tissue movements’ according to Gilbert [19]), (2) decreased inductive capacity of the altered blastema, and (3) disturbance of further differentiation of the organ anlage [25]. These investigations that continued the Spemann–Mangold experiments on organizers [29] also used experimental animals and revealed that hypoxia must occur before the onset of the formation of the organ anlage to induce severe developmental deviation. These findings found by hypoxia in amphibians and chicks are also fundamentally valid for mammals [30] and can be extrapolated to humans as well [25]. The effect of chronic hypoxia on some cardiac parameters was compared in rodents (rats) acclimatized either from the 4th day or the 12th week of postnatal life. PAH and right ventricular enlargement were found in both age groups [31]. The young hypoxic animals showed an increase in the weight of the right ventricle with a linear tendency with the pressure values of the right ventricle, while adult high-altitude exposed rats did not demonstrate such a relationship. Moreover, high altitude induces a significant increase in collagenous proteins with collagen I and III in young animals and collagen III only in the adult ones [31]. In comparing with the cardiovascular defects encountered at high altitudes, e.g., PAH, PDA, ASD, and VSD, it is impressive that these abnormalities can also be seen in experimental animals which have been used in a hypoxia-related environment. PAH is a vasculopathy of the pulmonary circulation characterized by arterial obliteration secondary to unchecked pathologic angiogenic processes. PAH is characterized by high circulating CD34 positive, CD133 positive proangiogenic progenitors, and endothelial cells that have a pathologic expression of HIF-1α [32]. Human urotensin II (U-II) is a cyclic vasoactive peptide composed of 11 amino acids with a structure similar to somatostatin [33,34]. The human form of U-II (hU-II) has been identified as an endogenous ligand for the G-protein-coupled receptor GPR14, also re-labeled as U-II receptor. Both hU-II and its receptor are intriguingly expressed in different cardiac and extracardiac tissues, including the brain, kidney, smooth muscle, and endothelium. It is known that hU-II is among the most potent vasoconstrictor peptides identified, with a potency greater than that of endothelin-1. The hU-II is a potent activator of reactive oxygen species (ROS) generation by reduced nicotinamide adenine dinucleotide phosphate (NADPH) oxidase in PA smooth muscle cells (PASMCs), leading to redox-sensitive activation of mitogen-activated protein kinases (MAPK) and Akt and subsequently to enhanced PAI-1 expression and increased proliferation. The hU-II may play an important role in pulmonary hypertension by promoting remodeling processes via activation of NADPH oxidases [33,34].

There is a higher risk for structural CHD in twin pregnancies, and the prevalence of CHD is 2% in otherwise uncomplicated monochorionic diamniotic (MCDA) gestations and 5% in cases of the twin–twin-transfusion syndrome (TTTS), particularly among recipient twins. Some hypotheses have been formulated, and theories have been based on abnormal placentation that occurs in monochorionic twins, particularly in cases that develop TTTS contributing to abnormal fetal heart formation [35,36]. The twinning process itself could lead to cardiac defects, but also the division of the fertilized ovum could be teratogenic. Moreover, early hypoxia, damage of the inner cell mass and the zona pellucida, delayed fertilization time, and slow tubal transport are possible causes of monozygotic twinning, which may also affect the development of the embryos. After segmentation, at least one major body axis in each embryo needs to rearrange, and this event is prone to errors, which might explain the higher prevalence of midline and laterality defects in monozygotic twins (e.g., cloacal exstrophy, anal atresia, anencephaly, spine defects, and CHD). Springer et al. [37] evaluated the prevalence of CHD in a large unselected cohort of monochorionic twin pregnancies combining diagnoses of prenatal and postnatal echocardiography and autopsy results. The authors confirmed a high prevalence of structural CHD (5.5%) showing that structural CHD, as well as ventricular hypertrophy and cardiomegaly, occurred significantly more often in monochorionic twin pregnancies complicated by TTTS compared with fetuses without TTTS [37]. Single gene defects associated with isolated or non-syndromic CHD have been delineated. Mutations in *NKX2.5* lead to isolated ASDs with atrioventricular conduction delay, while mutations in *GATA4* (a family of transcription factors characterized by their ability to bind to the DNA sequence GATA), a zinc finger transcription factor known to interact with the NK2 homeobox 5 (NKX2.5), which have been linked to isolated ASDs without conduction system abnormalities. Moreover, a mutation in *GATA4* specifically disrupted an interaction with T-box transcription factor (TBX5) suggesting that mutations in any of these interacting transcription factors can lead to CHD [38]. In Figure 1 is shown an interaction panel of several proteins linked to CHD using version 11 of STRING, an online bioinformatic tool using several databases [39]. It may be postulated that HIF-1α is linked to bone morphogenetic protein 4 (BMP4) in this early hypoxic insult causing not acardiac fetuses, but fetuses with structural defects such as those identified in high-altitude pregnancies. HIF-1α-dependent up-regulation of BMP4 mediates hypoxia-induced increase in TRPC expression in PASMCs, and BMP4 interaction is key in determining CHD. In the panel of Figure 1, there is also FOXH1, which is Forkhead box protein H1 with the ability to bind SMAD2 (Mothers against decapentaplegic homolog 2). It activates an activin response element via binding the DNA motif TGT(G/T)(T/G)ATT. FOXH1 is essential for the development of the growth of the embryo and its anterior heart field [40,41].

**Figure 1:**
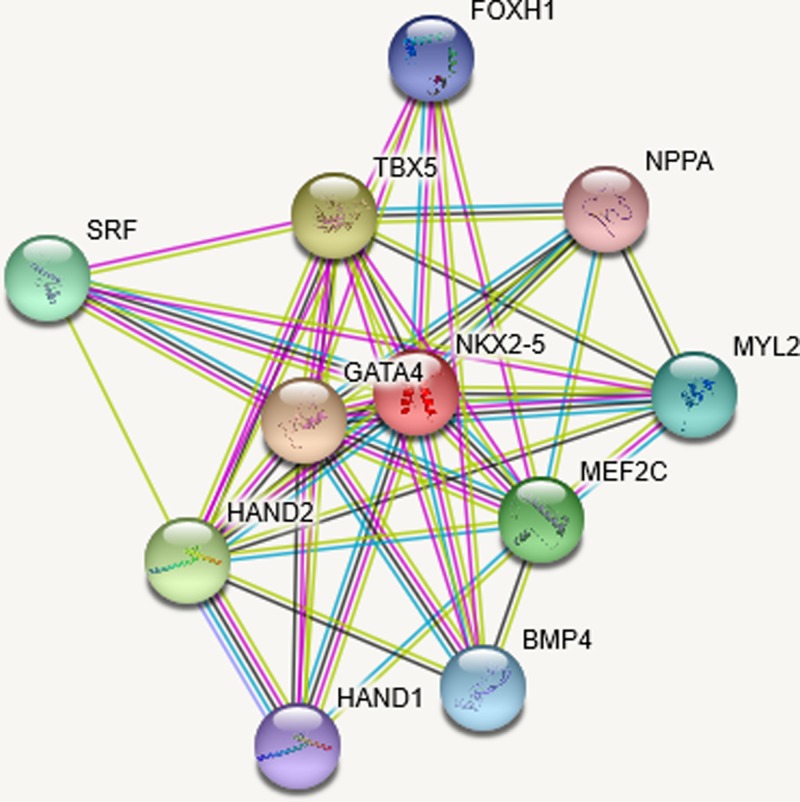
Splice isoforms or post-translational modifications are collapsed, while each node represents all the proteins produced by a single, protein-coding gene locus The edges represent protein–protein interactions with different color according to the interaction type. A red line indicates the presence of fusion evidence, green line a neighborhood evidence, blue line a cooccurrence evidence, purple line an experimental evidence, yellow line a textmining evidence, light blue line a database evidence, and black line a coexpression evidence.

Finally, it must be precisely known that children with CHD may have an intellectual impairment [42]. Neurodevelopmental outcomes are weakened in survivors of critical CHD in several developmental cerebral domains including motor, cognitive, and sensory outcomes. The cause of these neurodevelopmental deficits is multi-factorial and includes individual risk factors, cardiac anatomy, and cardiovascular physiology, brain development (e.g., myelination) as seen on magnetic resonance imaging (MRI). Despite early surgery, these shortfalls can extend into the adolescent and early adulthood years [42–45]. MRI studies have shown decreased total brain volume and white matter (WM) injury, which could be because of hypoxia [46]. The cellular/molecular mechanisms linked to brain immaturity and preoperative WM injury in newborns affected with CHD remain mostly unexplored. There is a rodent model of diffuse WM-injury exposing mice at neonatal age to chronic hypoxia, which showed alterations in oligodendrocyte development resulting in hypomyelination, including oligodendrocyte death, delayed differentiation of the oligodendrocytes, and, even, abnormal patterns of myelination [47,48]. Although the present study does not show encephaloclastic changes of early hypoxic damage, it is a well-established model to mimic hypoxic brain injury in premature infants [47–50]. In the future, it may be essential to evaluate whether the residents of high altitude also have such brain lesions or other micro- and macrostructural brain abnormalities [51]. There is a possibility that hypoxia could be a common factor responsible for intellectual impairment in children with CHD and high-altitude residents, and whether they might have similar cerebral lesions on high-quality MRI would be useful to know. The interaction between placenta and heart is just starting to be explored adequately and will deliver unconfutable data for the future development of the embryonic heart in the next decade [52].

In conclusion, Pan’s findings and my interactome analysis have important implications for signaling in CHD. New options may be accurately explored using current bioinformatic tools. Small groups may also be investigated to find mutations which could have an impact on finding pathways which have broader implications. The identification of mutations in multiple interacting cardiac developmental genes will be part of a routine screening of the nearest future that may be applied to our cardiac patients with CHD. This procedure may notably improve our understanding of the pathogenesis, promote surgical options, and ultimately improve the quality of healthcare of the 21st century.

## Abbreviations

ASD: atrial septal defect
BMP4: bone morphogenetic protein 4
CHD: congenital heart disease
EGLN1: endoglin 1
EPAS1: endothelial PAS domain-containing protein 1
HIF-1α: hypoxia-inducible factor 1 alpha
MRI: magnetic resonance imaging
NADPH: nicotinamide adenine dinucleotide phosphate
PA: pulmonary artery
PAH: pulmonary arterial hypertension
PASMC: pulmonary artery smooth muscle cell
PDA: patent ductus arteriosus
PHD2: prolyl hydroxylase domain 2
TRAP: twin-reversed arterial perfusion
TTTS: twin–twin transfusion syndrome
U-II: urotensin II
VEGF: vascular endothelial growth factor
VHL: Von-Hippel–Lindau
VSD: ventricular septal defect
WM: white matter
